# The association of depression, loneliness and internet addiction levels in patients with acne vulgaris

**DOI:** 10.1186/s13030-020-00190-y

**Published:** 2020-08-05

**Authors:** Coşkun Öztekin, Aynure Öztekin

**Affiliations:** 1 Department of Family Medicine, Faculty of Medicine, Hitit University, 19000 Çorum, Turkey; 2 Department of Dermatology, Faculty of Medicine, Hitit University, Çorum, Turkey

**Keywords:** Acne vulgaris, Loneliness, Depression, Internet addiction

## Abstract

**Background:**

Acne vulgaris is a very common skin disorder that has negative effects on the mood, self image and social relations of the patients. We want to evaluate the effects of acne vulgaris and its severity on depression, loneliness, internet addiction levels, and the quality of life of young adult females.

**Methods:**

Two hundred three female acne vulgaris patients and 202 healthy controls who admitted to the dermatology clinic of a university hospital formed the study sample. Global Acne Grading System (GAGS) was used to assess the severity of acne. The Young Internet Addiction Scale-Short Form (YIAS-SF), The Acne Quality of Life Scale (AQLS), The University of California Los Angeles-Loneliness Scale (UCLA-LS), and The Beck Depression Inventory (BDI) were used to collect information about the patients.

**Results:**

The median BDI and the mean UCLA-LS and YIAS-SF scores were higher in the Acne group than those in the control group. The correlations between acne severity and the 3 scales were not significant but the correlations between AQLS and BDI, UCLA-LS, and YIAS-SF were highly significant. In multiple regression analysis, age and the BDI score predicted the YIAS-SF score significantly.

**Conclusions:**

Our findings support the previous findings that acne vulgaris patients are prone to depression and loneliness, and expand these findings to the vulnerability against internet addiction. Loneliness and depression should be assessed and, if found, targeted by psychological means to prevent internet addiction in acne vulgaris patients.

## Introduction

The skin which is the most prominent and visible part of human body is very important for perceived attractiveness especially in youngs. Acne vulgaris is a very common skin disorder which affects up to 80% of adolescents and up to two-thirds of adults [1]. Evidence suggests that acne has an adverse effect on the self image of young people and may have negative effects on social relations [2]. Even moderate acne vulgaris may be a potential barrier for social relationships due to both social anxiety in these patients and for prejudices against them [3]. This may lead to loneliness which may be associated with low self-esteem, depression, smoking, alcohol abuse, obesity, and suicide [4].

There is not a one-way relationship between acne and stress. Stressful life events like university entrance exams or getting married have been shown to increase acne lesions [5]. Several psychoneuroendocrinological and psychoimmunological mechanisms have been proposed to explain the relationship between acne and stress [6]. The increased levels of corticotropin-releasing hormone, adrenal androgens and glucocorticoids during stress may have negative effects on cutaneous permeability, epidermal lipid synthesis, and antimicrobial defense [7]. In addition, neuroactive subsctances incuding substance P may stimulate sebaceous gland proliferation which may aggravate acne lesions [8].

The negative effects of acne vulgaris on the health-related quality of life in acne patients have been reported [9]. Effects on health-related quality of life are not homogenous; for example, females are affected more than males [10]. Some studies found greater impairment in the quality of life with greater acne severity [11] while such a relation could not be observed in others [12].

Isotretinoin is a retinoid medication used for the severe and difficult-to-treat forms of acne vulgaris [13]. The use of isotretinoin has been frequently associated with depression and less frequently with suicide and psychosis [14]. Isotretinoin may disrupt the functions of several cerebral regions like hippocampus, corpus striatum, and frontal cortex, particularly the orbitofrontal cortex, which are associated with the pathophysiology of depression [15].

Loneliness may be defined as the lack of social relations required by an individual or the low quality of relations perceived by an individual due to the lack of closeness, intimity and emotionality [16]. High level of loneliness is known to be present in adolescents with acne like other dermatological disorders [17].

In the last 2 decades, the explosive growth of the Internet use increased research activities about the addictive potential of the Internet [18]. Like other addictive disorders, internet addiction disorder includes several criteria such as preoccupation with the internet, withdrawal symptoms, tolerance, unsuccessful attempts to control internet use, continued excessive internet use despite the knowledge of negative psychosocial problems, the loss of interests, previous hobbies, entertainment as a result of, and with the exception of, internet use, the use of the internet to escape or relieve a dysphoric mood and deception of family members, therapists, or others [19]. Internet addiction is known to be more prevalent in individuals with inhibitory interpersonal relationship style and loneliness [20].

The aim of this study was to evaluate the effects of acne vulgaris and its severity on depression, loneliness and internet addiction levels, and the quality of life of young adult females who were in a very sensitive period for body image and social relations. Our hypotheses were that acne patients were lonelier and more addicted to internet than the controls. We proposed that patients who had more severe acne or whose life quality were more affected by the disease had higher levels of loneliness and internet addiction as well.

## Methods

### Participants

This study included adult female patients above 18 years of age who admitted to the dermatology clinic of Hitit University between June 2018 and March 2019 and who were diagnosed as acne vulgaris. The questionnaire forms were obtained from patients who were informed about the study and accepted to participate in the study before completing the questionnaire forms. Patients whose education level was less than primary school and who had cognitive dysfunctions at a level that prevented filling the questionnaires were excluded. Any treatment for a psychiatric disorder in the last 6 months and medical comorbidities were also in the exclusion criteria. Patients with acne vulgaris did not take any treatment for acne in the last 3 months although earlier treatments were not taken as exclusion criteria. The control group included healthy hospital staff and their relatives above 18 years of age who had no systemic, dermatological or psychiatric illness and who were graduated at least from primary school. Informed consent was obtained from all participants.

### Measures

#### Global acne grading system (GAGS)

This system is used to determine the severity of acne, the distribution of acne lesions in the body, and the lesion type. The total score ranges between 0 and 44. The patients can also be graded as no acne (0 points), mild severity (1–18 points), moderate severity (19–30 points), severe (31–38 points), and very severe (> 39 points) [21].

#### Young internet addiction test- short form (YIAT-SF)

This 12-item scale was developed by Pawlikowski et al. [22] from the 26 item Internet Addiction Scale which was developed by Young [23]. The validity and Reliability of the Turkish version of this scale was performed by Kutlu et al. [24].

#### Acne quality of life scale (AQLS)

This scale measures the effect of acne on the quality of life of patients [25]. The validity and reliability study of the Turkish version of this scale was performed by Demircay et al. [26]. The quality of life decreases with the increasing scores in this scale.

#### University of Carolina los Angeles loneliness scale (UCLA-LS)

The original UCLA-LS was developed by Russel et al. [27]. In this study, we used a shorter version of this scale which was revised by Eskin (2001) [28]. Higher scores in this scale correspond to higher levels of loneliness.

#### Beck depression inventory (BDI)

This scale was developed by Beck et al. [29]. It consists of 21 items and is scored from 0 to 3. The validity and reliability study of its Turkish version was performed by Hisli (1988) [30].

### Procedure

All subjects included in this study filled sociodemographic data form, BDI, UCLALS, and IAS. The severity of acne was assessed by GAGS. Acne disease duration and previous treatments they received were also recorded.

### Statistical analysis

Mean ± standard deviation or median (interquartile range) were used to summarize descriptive statistics. The Kolmogorov-Smirnov test was used for the normality testing of numeric variables. The comparisons between the acne and the control groups were performed with Independent Samples T test when the variables were normally distributed and with the Mann-Whitney U test otherwise. Since age and education were significantly different between the groups, their effects were controlled in the comparisons for BDI, UCLA-LS, and YIAS-SF. Robust ANOVA was used for non-parametric comparisons. The results were evaluated using Raov function in the Rfit package of R program. Pearson Chi-Square test was used for the comparison of differences between the groups. The correlations between scale scores were performed with Pearson correlation when the distribution was normal and with Spearman correlation otherwise. Bonferroni correction was used for multiple testing before the evaluation of the results. The effects of several predictors on YIAS were evaluated with simple and multiple linear regression models. Jamovi (version 0.9.6.9) and R 3.6.0 package programs were used for statistical analyses, and the significance level was accepted as 0.05.

## Results

This study included 203 female patients with acne vulgaris and 202 control cases. The mean age of acne patients (21.0 ± 2.8) was significantly lower than that of the controls (22.1 ± 3.9) (*p* = 0.001). University students and graduates were more common in the Acne group. The median BDI (12.0 vs 8.0), and the mean UCLALS (36.8 ± 10.5 vs 32.3 ± 9.2) and YIAS-SF (26.1 ± 8.1 vs 24.3 ± 8.1) scores were higher in the Acne group than those in the control group (*p* < 0.001, *p* < 0.001, and *p* = 0.026, respectively). We controlled these results for age and education variables. BDI and YIAS-SF were still significant while UCLA-LS was not (Table 1).

**Table 1:** Sociodemographic characteristics and scale scores of the acne vulgaris patients and the controls

	Group			
	Acne (***n*** = 203)	Control (***n*** = 202)	p	Covariate Age
**Age**	21.0 ± 2.8	22.1 ± 3.9	**0.001**	–
**Marital Status**				
Married	20 (9.9%)	33 (16.3%)	0.074	–
Single	183 (90.1%)	169 (83.7%)		
**Education Status**				
Primary School	7 (3.4%)	10 (5.0%)	**0.044**	–
Secondary School	11 (5.4%)	15 (7.4%)		
High School	73 (36.0%)	94 (46.5%)		
University	112 (55.2%)	83 (41.1%)		
**BDI, median [IQR]**	12.0 [6.0. 20.5]	8.0 [3.0. 14.0]	**< 0.001**	**0.019**^a^
**UCLALS**	36.8 ± 10.5	32.3 ± 9.2	**< 0.001**	0.703^b^
**YIAS-SF**	26.1 ± 8.1	24.3 ± 8.1	**0.026**	**< 0.001**^b^

*BDI* Beck Depression Inventory, *IQR* Interquartile range, *UCLALS* University of California Los Angeles Loneliness Scale, *YIAS-SF* Young Internet Addiction Scale- Short Form

^a^: Non parametric ANCOVA was used

^b^: ANCOVA was used

According to GAGS, the severity of acne in patients in our study was mild (*n* = 106), or moderate (*n* = 97). No patient had severe or very severe acne. Mean disease durations were similar between these two groups (Table 2).

**Table 2:** Number of patients in mild and moderate acne severity groups according to GAGS and their mean disease durations

	Acne Severity	
	Mild (GAGS:1–18) (***n*** = 106)	Moderate (GAGS:19–30) (*n* = 97)
**Mean GAGS**	13.95 ± 3.30	23.03 ± 3.27
**Mean disease duration (months)**	56.71 ± 34.49	58.21 ± 36.07

*GAGS* Global Acne Grading System

The correlations among scale scores and several disease parameters in the acne vulgaris patients were calculated using Spearman correlation analyses. Significant correlations were found between AQLS and BDI (r = 0.583, *p* < 0.001), AQLS and UCLA-LS (r = 0.459, *p* < 0.001), AQLS and YIAS (r = 0.278, *p* < 0.001), and UCLA-LS and YIAS (r = 0.277, *p* < 0.001) (Table 3). We also performed univariate and multiple regression analyses to understand the factors that affected YIAS score. In univariate analysis AQLS, BDI, and UCLA-LS scores and age were found to affect YIAS score significantly. In multiple regression analysis, only age (β = − 0.53, *p* = 0.005) and BDI (β = 0.17, *p* = 0.023) were significant.

**Table 3:** Correlation between several parameters in the acne vulgaris group

			Correlation	
Variables			r	***p***
**Acne Severity**	**–**	**AQLS**	0.034	0.625
**Acne Severity**	**–**	**BDI**	0.040	0.568
**Acne Severity**	**–**	**UCLA Loneliness Scale**	0.018	0.800
**Acne Severity**	**–**	**YIAS**	0.047	0.510
**AQLS**	**–**	**BDI**	0.583	**< 0.001**
**AQLS**	**–**	**UCLA Loneliness Scale**	0.459	**< 0.001**
**AQLS**	**–**	**YIAS**	0.278	**< 0.001**
**UCLA Loneliness Scale**	-	**YIAS**	0.277	**< 0.001**

*AQLS* Acne Quality of Life Scale, *BDI* Beck Depression Inventory, *UCLA* University of California Los Angeles, *YIAS* Young Internet Addiction Scale

165 patients (77.47%) received previous treatments. None of the acne severity, AQLS, BDI, UCLA-LS, and YIAS scores were different between patients who received or did not receive previous treatments (data not shown). Twenty four patients (11.8%) used isotretinoin previously. Their AQLS, BDI, UCLA-LS, and YIAS scores were tended to be higher than the patients’ who had not used isotretinoin, but the results were not statistically significant (Table 4).

**Table 4:** Comparison of several parameters according to previous isotretinoin use of the patients

	Previous isotretinoin use		
	No (***n*** = 179)	Yes (***n*** = 24)	***p***
**Age**	21.0 ± 2.9	21.3 ± 2.4	0.511
**Disease duration**	55.6 ± 35.4	71.0 ± 31.2	**0.033**
**Acne severity**	18.3 ± 5.6	18.5 ± 6.1	0.857
**AQOL**	15.9 ± 5.6	17.4 ± 4.8	0.171
**BDI (median [IQR])**	12.0 [6.0–20.0]	16.0 [9.8–21.0]	0.143
**UCLA-LS**	36.6 ± 10.7	38.8 ± 9.0	0.281
**YIAS**	25.8 ± 8.1	27.9 ± 8.4	0.263

*AQOL* Acne Quality of Life Scale, *BDI* Beck Depression Inventory, *IQR* Interquartile Range, *UCLA-LS* University of California Los Angeles Loneliness Scale, *YIAS* Young Internet Addiction Scale

In 101 (49.75%) patients acne involved only face and in 102 (50.25%) patients acne involved both face and body. No difference was found in acne severity, AQLS, BDI, UCLA-LS, and YIAS scores according to the location of acne.

## Discussion

Although dermatological disorders like acne vulgaris are not life-threatening, they are very important in that they alter appearance; therefore, they may affect the psychosocial status, daily activities, relationships and quality of life of the individuals [31]. Our study expands these findings and adds findings about internet addiction in acne vulgaris patients.

Acne usually affects adolescents but its negative effects on individual’s psychological status are not limited to this period [32]. Hassan et al. [33] investigated acne patients at or above 20 years of age and found that their self awareness level was higher than that of 16–19-year-old patients. Also, Lasek and Chren (1998) [34] found that the rate of patients who were most bothered by the appearance of acne were higher in 30–39-year-old patients. The mean age of our patient group was 21.0 which was a risky period for the adverse psychological effects of acne vulgaris.

An association between acne vulgaris and depression is known for a long period of time [35]. Depression was found to be 2–3 times more prevalent in acne patients than in the general population, and twice prevalent in males than in many female acne patients who reported to have depression [36]. We also found higher depression levels in our female acne vulgaris patients compared with healthy the controls. The presence of acne, rather than its severity, may cause psychological symptoms in acne patients. Many previous studies could not find a relationship between psychological symptoms like depression, anxiety, or social stress and acne severity [37, 38]. We also could not find a relationship between acne severity and depression scores. This finding may suggest that the presence of acne, rather than its severity, may cause psychological symptoms in acne vulgaris patients. On the other hand, this may reflect the fact that patient’s perception of acne severity may not coincide with the clinical severity assessment with GAGS [39].

Acne treatment may have various effects on the psychological status of acne patients. Their moods may improve with a successful treatment of acne vulgaris [40]. However, isotretinoin treatment is known to induce depression and anxiety [41]. In our study, patients who had been previously treated with isotretinoin had higher depression, life quality, and internet addiction scores although the results were not statistically significant. As our study was cross-sectional and our patients were not taking a treatment for at least 3 months before the study, we could not estimate the effect of isotretinoin treatment on depression, life quality, or internet addiction. Besides, isotretinoin treatment is generally preferred in severe acne patients, which may also be a confounder of these findings.

The mean quality of life score in our patients (16.06 ± 5.54) was higher than those in the previous studies in Turkey with the same questionnaire like those of Demircay et al. (13.5) [26] and Unal et al. (13.58 ± 4.84) [42]. The samples in these studies were younger than those in our study and were male-female mixed groups unlike our study which included only females. These findings suggest that the life quality of young adult females affected more by acne vulgaris than adolescents or males possibly because of their increased self awareness in accordance with a previous study by Ismail et al. [43].

Social withdrawal and social phobia have been frequently reported in acne vulgaris patients [44]. Their self-esteem is known to be low as well [4] and they percieve higher levels of stigmatization related to their skin than people without acne [45]. These factors may cause avoidance from social interactions and may lead to loneliness. We found higher level of loneliness just like a previous study performed in adolescents [17]. This finding is understandable considering the social inhibition experienced by acne vulgaris patients.

Internet usage was limited to a small group of businessman or academic people 20–25 years ago but today children and young people in all modern societies also use internet in their daily lives. Internet addiction can be defined as excessive use of internet; inability to resist the desire to use it; extreme nervousness when deprived of it; and deterioration in business, social, and family life due to excessive internet use [46]. We found a higher level of internet addiction in our patients compared with the controls. The individuals who feel alone and have difficulties in social life may use internet for longer periods because they feel more comfortable in the virtual environment and may establish new friendships in social media. A previous study in Turkey found higher levels of problematic internet use in the adolescents with acne vulgaris compared with the controls [47]. We tried to determine the factors leading to internet addiction in our young adult acne vulgaris patient sample. We found a significant correlation between loneliness and internet addiction. The results of multiple logistic regression analysis revealed that internet addiction probability was higher in younger patients with high BDI scores. It is known that internet addiction is more common in younger people [48]. Also, a two-way relationship is suggested between internet addiction and depression in which both conditions augment the severity of each other [49]. Thus, it can be concluded that higher depression levels in acne vulgaris patients may cause higher levels of internet addiction. As previously mentioned, acne vulgaris is known to decrease self-esteem especially in young women and may cause a perception of social rejection [4, 50]. Such perceived or actual social rejection may diminish the desire for peer interactions which may lead to loneliness. And a lonely youth will be more prone to spend much time in internet and develop internet addiction. A previous study in Turkey also found that loneliness had a direct effect on Internet addiction [51].

There are some limitations of this study. First, the use of self-report scales prevents talking about exact psychiatric diagnoses in these patients. The complaints in the self-report scales may be augmented by the subjects or may fail to reflect the actual condition of the subject. Second, due to cross-sectional design of the study, a causal relationship cannot be established. A longitudinal study design including the data of the patients before and after the onset of acne vulgaris may eliminate baseline differences and more reliably demonstrate the psychiatric effects caused by acne vulgaris. Third, this study included only female patients, which made it impossible to have estimates about male acne patients.

## Conclusions

In conclusion, this study repeats the previous findings that acne vulgaris patients have many psycho-social problems and extends these findings to internet addiction which is a very important public health problem in modern societies today. Dermatologic interviews with female acne vulgaris patients may include assessments for the symptoms of loneliness, depression, and internet addiction and patients with psychological symptoms may be consulted to a psychiatrist in order to provide a more holistic treatment. The effects of such multidisciplinary approaches towards both the dermatological and psychological symptoms of the patients may be investigated in future research.

## Data Availability

Not applicable.

## Abbreviations

GAGS: Global Acne Grading System
YIAS-SF: Young Internet Addiction Scale-Short Form
AQLS: Acne Quality of Life Scale
UCLA-LS: The University of California Los Angeles-Loneliness Scale
BDI: Beck Depression Inventory
